# Functional MRI evidence for the decline of word retrieval and generation during normal aging

**DOI:** 10.1007/s11357-015-9857-y

**Published:** 2015-12-28

**Authors:** M. Baciu, N. Boudiaf, E. Cousin, M. Perrone-Bertolotti, C. Pichat, N. Fournet, H. Chainay, L. Lamalle, A. Krainik

**Affiliations:** 1 Univ. Grenoble Alpes, LPNC, F-38040 Grenoble, France; 2 CNRS, LPNC UMR 5105, F-38040 Grenoble, France; 3 Univ. Savoie Montblanc, LPNC, F-73000 Chambéry, France; 4 Laboratoire d’Etude des Mécanismes Cognitifs, Université Lumière Lyon 2, F-69676 Bron, France; 5 UMS IRMaGe CHU Grenoble, Univ. Grenoble Alpes, F-38043 Grenoble, France; 6 GIN Univ. Grenoble Alpes, F-38043 Grenoble, France

**Keywords:** Normal aging, Lexical, Executive, Semantic, Fluency, fMRI

## Abstract

This fMRI study aimed to explore the effect of normal aging on word retrieval and generation. The question addressed is whether lexical production decline is determined by a direct mechanism, which concerns the language operations or is rather indirectly induced by a decline of executive functions. Indeed, the main hypothesis was that normal aging does not induce loss of lexical knowledge, but there is only a general slowdown in retrieval mechanisms involved in lexical processing, due to possible decline of the executive functions. We used three tasks (verbal fluency, object naming, and semantic categorization). Two groups of participants were tested (Young, Y and Aged, A), without cognitive and psychiatric impairment and showing similar levels of vocabulary. Neuropsychological testing revealed that older participants had lower executive function scores, longer processing speeds, and tended to have lower verbal fluency scores. Additionally, older participants showed higher scores for verbal automatisms and overlearned information. In terms of behavioral data, older participants performed as accurate as younger adults, but they were significantly slower for the semantic categorization and were less fluent for verbal fluency task. Functional MRI analyses suggested that older adults did not simply activate fewer brain regions involved in word production, but they actually showed an atypical pattern of activation. Significant correlations between the BOLD (Blood Oxygen Level Dependent) signal of aging-related (A > Y) regions and cognitive scores suggested that this atypical pattern of the activation may reveal several compensatory mechanisms (a) to overcome the slowdown in retrieval, due to the decline of executive functions and processing speed and (b) to inhibit verbal automatic processes. The BOLD signal measured in some other aging-dependent regions did not correlate with the behavioral and neuropsychological scores, and the overactivation of these uncorrelated regions would simply reveal dedifferentiation that occurs with aging. Altogether, our results suggest that normal aging is associated with a more difficult access to lexico-semantic operations and representations by a slowdown in executive functions, without any conceptual loss.

## Introduction

Unlike other cognitive processes sensitive to aging such as executive functions, memory, and information processing speed (Salthouse 2009), language abilities remain stable longer over the lifespan, even improving in terms of vocabulary, semantics, and speech processing (Kavé et al. 2009, Salthouse 2009, Verhaegen and Poncelet 2013). Moreover, overlearned automatic processing, word recognition, and semantic skills remain unaffected by age (Burke and MacKay 1997; Shadden 1997). Although language seems to be globally intact in older adults (Meyer and Federmeier 2010) more detailed analyses indicate that older adults are slower to access meaning and conceptual representations (Huang et al. 2012) and to produce language (see review in Stine-Morrow and Shake 2009). Indeed, people over 65 could experience impairment in retrieving and generating words (Feyereisen 1997; Evrard 2002; Burke and Shafto 2004; Gollan and Brown 2006) with lower efficiency of lexical production. Although the mechanisms explaining this language difficulty are not completely understood, two main explanations may be considered: (a) a general decline in executive functions which are non-specific to language or (b) a deficit in accessing the specific levels of language processing.

According to the first explanation, a word generation deficit would be induced by the decline in executive functions (Craik and Byrd 1982) and/or processing speed (Salthouse 1996) as a result of aging-related anatomical reductions within the frontal lobes (Raz et al. 2007; Ullman and Pierpont 2005; West 2000). The decline of frontal functioning with the slowdown in executive functions with aging can be revealed by tasks that are highly dependent on the cognitive control (West 2000) or attention (Dennis and Cabeza 2008).

According to the second explanation considering the direct lexical production decline, the impairment of the specific stages of language production described by the psycholinguistic models might occur. Overall, the psycholinguistic models of lexical production (Caramazza 1997; Dell and O’Seaghdha 1992; Levelt 1992) consider three main stages of word retrieval and generation, i.e., conceptual (lexical), meaning (lexical retrieval, semantic), and phonological–phonetic (for details, see Indefrey 2011). The anatomical network specific to each of these stages was also identified (see for details, Indefrey and Levelt 2004; Indefrey 2011).

Regarding aging, Wierenga et al. (2008) suggested that language difficulties can be determined by a dysfunction at three possible levels: (a) retrieval abilities with search for meaning, cognitive control, and selection processes in order to access and retrieve the lexical forms (processes depending on frontal regions) (Braver and Barch 2002) (b) semantic associations, which are mainly dependent on the inferior temporal (fusiform) gyrus (Ishai et al. 1999; Wierenga et al. 2008) and (c) phonological word form, explained by a reduction of information transmission from lexical to phonological representations (Burke and MacKay 1997; MacKay and Burke 1990) associated with atrophy of the left insula (Shafto et al. 2010). Wierenga et al. (2008) showed that for an accuracy level equivalent to younger adults, older participants recruit more strongly the frontal cortex but not the inferior temporal regions, suggesting that aging is rather associated with deficits of retrieval mechanisms instead of a loss of stored conceptual representations (Kemper et al. 2001; Wierenga et al. 2008). Furthermore, the authors correlated the BOLD (Blood Oxygen Level Dependent) signal measured in the frontal regions with the behavioral scores. Based on results, they concluded for compensatory mechanisms recruited by older adults in order to maintain a correct level of task performance.

The aim of this fMRI study was to assess the effect of normal aging on word retrieval and generation by comparing two groups of healthy, cognitively unimpaired participants, young and older, and by using a multi-task and multimodal approach (behavioral, neuropsychological, and neurophysiological/fMRI). Our main hypothesis was that normal aging is not associated with loss of concepts and does not alter the lexical conceptual level but mostly relies on the dysfunction of retrieval abilities with a more difficult searching of semantic and phonological representations, as well as of semantic associations. We used a panel of tasks instead of a unique task, to recruit and map, as completely as possible, processes and cerebral networks, respectively, that are involved in word retrieval and generation. Indeed, participants performed three tasks: (a) verbal (semantic, categorical) fluency (VF), (b) object naming (ON), and (c) semantic categorization with Pyramids Palm Tree Test (PPTT) (Howard and Patteron 1992). The choice of these tasks was based on (a) their ability to map the word generation network using a differential recruitment of linguistic processes that might be sensitive to normal aging: *retrieval* (RET) of word form representations (semantic, phonological) based on executive functions (selection, flexibility, cognitive control) recruited mainly by the verbal fluency (VF) task, mapping *lexico-semantic processes and associations* (SEM) recruited mainly by the object naming (ON) and *both RET and SEM* associated with *conceptual access* (CON) for the semantic categorization (PPTT) task; (b) their use in clinical neuropsychology practice for the language testing. Typically, VF is sensitive to aging (Brickman et al. 2005; Pasquier et al. 1995) and recruits fronto-temporal regions (Gleissner and Elger 2001; Martin et al. 1994; Pihlajamaki et al. 2000). This task relies on the interaction between language and executive functions and is frequently used to assess the integrity of strategic processes of word retrieval (Benton 1968). The cerebral regions activated by VF reflects RET processes to access lexical storage and word meaning, selection and switching between items and categories, and phonetic encoding before articulation and overt speech. Furthermore, the temporary inability to find known words (tip-of-the tongue states, TOT; Gollan and Brown 2006) corresponds to a slowdown in *Naming*, according to the Boston Naming Test (Kaplan et al. 1983) which justifies the use of ON task in our study. Additionally, using a non-invasive brain stimulation (NIBS) approach, Cotelli et al. (2012) underlined that left frontal and temporal areas are crucial for naming. Overall, object naming engages large neural networks (Price et al. 2005) which are sensitive to aging (Wierenga et al. 2008). Finally, beyond its ability to explore word retrieval and generation, the use of PPTT is pertinent to access the semantic memory (Mummery et al. 1998) and this test is particularly sensitive in pathological aging (semantic dementia) associated with asymmetric atrophy of the anterior temporal lobes, anomia, deterioration of expressive and receptive vocabulary and of semantic memory (Hodges and Patterson 2007).

According to our main hypothesis regarding the lack of conceptual loss and the difficulties to access levels of word processing, the behavioral results should indicate a similar accuracy level between groups, but with longer latencies in older adults compared to the young. At a cognitive level, the neuropsychological scores for executive functions should be significantly lower in older adults compared to the younger. Moreover, the older adults should not be impaired for vocabulary tests and for overlearned semantic information, given their greater linguistic experience with age. At the cerebral level, we expect that older adults show for word retrieval and generation, a pattern of cerebral activation, which is not only a weaker version of what is observed in younger individuals but is also atypical at an intra- and inter-hemispheric level. Additionally, correlations between the BOLD signal measured in aging-related regions and behavioral and cognitive scores for each participant will allow us to determine the role of regions recruited by the older adults to perform the tasks, as well as the possible compensatory mechanisms underlying these atypical patterns.

## Material and methods

### Participants

Thirty participants were comprised in two groups: Young group, YG (*N* = 16; five females, Min = 30 years, Max = 59 years, *M* = 42.6 years, SD = ±9.5 years) and Aged group, AG (*N* = 14, four females, Min = 60 years, Max = 84 years, *M* = 72.2 years, SD = ±6.1 years). Other inclusion criteria were the absence of cognitive impairment (as assessed by the *Mini Mental State Examination*, MMSE) and the absence of psychological anxiety and depression (as assessed by the *Hospital Anxiety and Depression scale*, HAD). Additionally, we checked that participants did not have objective episodic memory deficits (“5 words” of Dubois test). All participants were native French speakers and were highly educated, according to the Poitrenaud questionnaire (Kalafat et al. 2003). They were right handed, according to the Edinburgh Handedness Inventory (Oldfield 1971). and had normal or corrected-to-normal vision (Table 1). They were recruited through ads diffused via associations of older adults and via the web. All participants gave informed written consent for the experiment. The local Ethics Committee approved this study (CPP N°: 2014-A00569-38), which was in accordance with the Code of Ethics of the World Medical Association (Declaration of Helsinki) for experiments involving humans.

**Table 1:** Demographic information of participants in terms of age, gender, handedness (Edinburgh inventory), as well as cognitive (MMSE), psychiatric (HAD), and episodic memory (5wD) scores

Characteristics	Groups				*T* test (ddl = 28)	*P* value
	Young Group		Aged Group			
Gender ratio (M/F)	11/5		10/4			
	Mean	s.d.	Mean	s.d.		
Age	42.6	9.5	72.2	6.1	9.94	<0.001*
Edinburgh Scale	89.2	23.1	93.4	12.7	0.59	0.55
ESC	4	0	3.8	0.3	1.57	0.13
MMSE	29.1	1.5	29	1.2	0.23	0.82
HAD—anxiety	6	2.2	6	1.6	0.00	1
HAD—depression	2.25	1.5	3.7	2.7	1.92	0.06
EM (5wD)^d^	9.93	0.25	9.92	0.26	0.26	0.9

Statistical values for comparisons between groups (young, aged) are mentioned. With the exception of age, no other significant difference was obtained for these values

*ESC* Education and SocioCultural level, *MMSE* Mini Mental State Examination, *HAD* Hospital Anxiety and Depression scale, *EM* Episodic Memory, *(5wD)* (5 words Dubois), *s*.*d*. standard deviation

### Neuropsychological assessment

The objective of this assessment was twofold: to (a) screen for any cognitive deficits and (b) evaluate specific cognitive domains and correlate participants’ behavior and fMRI data (BOLD signal) with the cognitive scores to further understand possible aging effects on lexical generation and processing. The neuropsychological tests were classified into two main categories:

- Tests for inclusion criteria (Cognitively unimpaired, without anxiety or depression)

(a) General cognitive level: the MMSE (Folstein et al. 1975, see Kalafat et al. 2003 for the French version) is a sensitive, valid, and reliable questionnaire for detecting cognitive impairment (cutoff value was 25); (b) Episodic memory: the *5wD* (Dubois et al. 2002, Vellas and Michel 2002) screens for learning and episodic memory deficits; (c) Psychiatric level: the HAD (Zigmond and Snaith 1983) evaluates levels of anxiety and depression.

- Tests for specific cognitive domains

(a) Vocabulary and verbal intelligence: *Mill*–*Hill vocabulary scale* (Deltour 1993) measures the *verbal intelligence level* and requires explaining the meanings of words and selecting the correct synonym for each word from a list; (b) Verbal fluency: *Categorical* (semantic) *Fluency test* (Cardebat et al. 1989) evaluates the integrity of lexico-semantic store, strategic processes for lexical searching and retrieval, and integrity of phonetic and articulatory processes; (c) Executive functions: *Trail Making test* part A (TMT-A) and part B (TMT-B) (Tombaugh 2004) measures visual search speed, scanning, processing speed, and mental flexibility. The forward and backward recall sub-tests of the *Digit span Memory test* (Weschler 1997) evaluate short-term and working memory using verbal items. The *Frontal Assessment Battery* (FAB) (Dartinet and Martinaud 2005) globally evaluates executive functions (frontal efficiency); (d) Semantic memory: *Verbal Automatisms test* (Beauregard 1971) and *derived IQ test* are used specifically for determining the level of overlearned semantic information. The Verbal Automatisms test used in our study has consisted of reading by the experimenter, the beginning of several overlearned French expressions and then asking the participant to complete it (e.g., the French expression *La fourmi et la*…./*The Ant and the*… should be completed with the word *cigale*/*grasshopper*). The derived IQ was then calculated on the basis of Verbal Automatisms scores. This test is sensitive to aging and older adults generally show higher scores than the young adults. Additionally, the *McNair Questionnaire* (self-assessment of cognitive deficits) (McNair and Kahn 1983) evaluates, non-specifically and subjectively, the frequency of cognitive complaints (language, memory, etc.) in the daily life.

### Functional MRI assessment

Three tasks have been performed in three separate runs: (a) semantic verbal fluency (VF), (b) object naming (ON), and (c) semantic categorization with Pyramid Palm and Tree Test (PPTT). All three runs were block-designed and alternated tasks and control periods. All runs started with an activation task followed by a control condition. Participants provided verbal (oral) responses during VF and ON and manual responses during the PPTT task. Behavioral responses were recorded and analyzed in terms of accuracy (% correct responses, %CR) and reaction time (RT ms). E-prime software (Psychology Software Tools, Pittsburgh, PA) was used to implement stimuli into the three experiments and to record manual responses provided during PPTT. Oral responses provided during VF and ON were recorded via an MRI-compatible microphone (FOMRI™ III, version 1.2). All participants went through a short training session outside the scanner, using different items than those presented during the fMRI experiments.

#### Tasks, stimuli, and paradigm

- Semantic verbal fluency (VF)

During VF task periods, the participants were required to overtly generate as many words as possible belonging to a semantic category. Specifically, four activation periods for four categories (animals, clothes, vegetables, and sports) were presented, and each of them started with a visually presented word indicating the category of words to generate. Each activation period lasted 1 min. Activation periods alternated with control periods (each of them lasting 30 s), during which participants were required to fixate on a central cross and try to not generate words. Generated words were recorded via a microphone fixated to the coil. Words indicating the category of words to generate and the fixation were written in black Arial font size 40 on a white screen. The VF run duration was 6 min.

- Object naming (ON)

During ON periods, participants were required to overtly name pictures presented on the screen. Stimuli were black and white simple drawings of objects and animals included in the basic DO-80 (Metz-Lutz et al. 1991). Four task periods comprised the ON run. Each period lasted 50 s and 20 stimuli/period were presented (80 images in total). Task periods were separated by control periods, during which a gray square or round shape (same number and same duration as the task stimuli) were randomly presented. Participants had to simply say “square” or “round.” A 500-ms fixation separated the task and control stimuli. Oral responses were recorded via a microphone fixated to the coil. In terms of response recording, we calculated the %CR for this task and we measured the RT. The ON run duration was 7.06 min.

- Semantic categorization with Pyramid Palm and Tree Test (PPTT)

During PPTT (Howard and Patterson 1992) task periods, participants were required to perform a semantic categorization task with stimuli composed of three images each. A given stimulus was represented by a top image (target image) and two bottom images (choice images). Participants had to choose among the two bottom images the one that is the most semantically related to the target (top). Manual responses were provided with their right-dominant hand by pressing two buttons with the index (for left bottom image) and the middle finger (for right bottom image). Four task periods were included in the PPTT run. Each task period lasted 56 s and 13 stimuli/period were presented (52 stimuli in total). Task periods alternated with control periods, during which a visual matching task was required: based on shape, the top image (round or square shape) had to be matched visually with one of the two bottom images. Control periods had the same duration and number of trials as the task periods. Manual responses were recorded, the accuracy (%CR) was calculated, and RT was measured for each participant. The PPTT run duration was 9.06 min.

#### Functional MR acquisition

The experiments were performed in a whole-body 3 T MR scanner (Philips Achieva) with 40 mT/m gradient strength. For functional scans, the manufacturer-provided gradient-echo/T2*-weighted EPI method was used. Forty-four adjacent axial slices parallel to the bi-commissural plane were acquired in interleaved mode. Slice thickness was 3.5 mm. The in-plane voxel size was 2.3 × 2.3 mm (216 × 216 mm field of view acquired with a 72 × 72 pixels data matrix; reconstructed with zero filling to 128 × 128 pixels). For the three functional runs, the main sequence parameters were TR = 2.5 s, TE = 30 msec, and flip angle = 77°. Finally, a T1-weighted high-resolution three-dimensional anatomical volume was acquired, by using a 3D Modified Driven Equilibrium Fourier Transform (MDEFT) sequence (field of view = 256 × 224 × 176 mm, resolution 1.333 × 1.750 × 1.375 mm, acquisition matrix 192 × 128 × 128 pixels, reconstruction matrix 256 × 128 × 128 pixels).

### Data processing

#### Neuropsychological scores and behavioral data

All scores were situated within the norms. For each test, cognitive scores obtained for AG and for YG were compared using *t* tests. Behavioral scores during fMRI were recorded and analyzed using *t* tests separately for each task, in order to compare AG and YG and assess age effects. *t* tests were performed for %CR and RT (ms), except for the VF task, where the analysis was based on fluency scores only. Indeed, we did not calculate response latency for responses provided by participants inside the magnet during VF. However, despite the scanner noise, the quality of word recognition was not altered and we could measure the accuracy in terms of number of generated words (we checked that generated words belonged to the indicated category).

#### Functional MRI data

The general linear model (Friston et al. 1994; Friston et al. 1995) in SPM12 (Welcome Department of Imaging Neuroscience, London, UK, www.fil.ion.ucl.ac.uk/spm) implemented in MATLAB 7 (Mathworks Inc., Sherborn, MA, USA) was used. Each condition was modeled using a canonical hemodynamic function model. Data analysis started with the spatial preprocessing steps. T1-weighted anatomical volume was co-registered to mean images created by the realignment procedure and was normalized to the MNI space using a trilinear interpolation. Anatomical normalization parameters were subsequently used for the normalization of functional volumes. Finally, each functional volume was smoothed by an 8-mm FWHM (full width at half maximum) Gaussian kernel. Time series for each voxel were high-pass filtered (1/128 Hz cutoff) to remove low-frequency noise and signal drift. After spatial preprocessing steps, separate statistical analysis for each task was performed. For each task, the conditions of interest (Task, Control) were modeled as two regressors built as boxcar functions, convolved with a canonical hemodynamic response function (HRF). Movement parameters derived from the realignment corrections (three translations and three rotations) were also entered in the design matrix as additional factors. Furthermore, behavioral scores were entered as a parametric modulator in the model for PPTT and ON tasks. The general linear model was then used to calculate the parameter estimates of the activity for each voxel, each condition, and each participant. Statistical parametric maps were generated from linear contrasts between the HRF parameter estimates for the different experimental conditions. The spatial resolution of the statistical parametric maps was the same as the spatial resolution of the functional MR acquisition (2.3 × 2.3 × 3 mm). Specific effects were tested with the appropriate linear contrasts of the parameter estimates, and the corresponding contrast images were subsequently entered into a random effects analysis. First, we evaluated the task-related cerebral network using a one-sample *t* test (K > 5; *p* < 0.05 corrected; *T* = 5.9). Second, we evaluated the effect of age for each using a two-sample *t* test (*K* = 10, determined empirically, *p* < 0.001; *T* = 3.55). Brain regions activated from the statistical contrasts were identified and labeled using the macroscopic parcellation of the MNI single subject reference brain (Tzourio-Mazoyer et al. 2002). For each task and after selecting the ROI using MarsBar software (http://marsbar.sourceforge.net/), an in-house software allowed us to measure the variation of %MR signal variation in each ROI. The %MR (BOLD signal) values obtained for the ROI resulting from the contrast AG vs. YG (i.e., age-dependent regions) were subsequently included in a correlation analysis (Pearson correlation) with the behavioral and neuropsychological scores showing age-effect in AG. Finally, we calculated a classical lateralization index (Seghier 2008), LI, as the difference between the number of activated voxels (k) in the left hemisphere and in the right hemisphere divided by the sum of voxels in both hemispheres. Regions considered for the calculation of LI were those showing an age-effect (resulting from two-sample *t* tests) and that were deemed to have an essential role in word production, as previously mentioned in the “Introduction” section (Indefrey 2011).

## Results

### Neuropsychological scores and behavioral data

No differences between the groups were obtained for the general cognitive status (MMSE), psychiatric (HAD) and episodic memory (5wD) tests (see Table 2a). The groups were not different in terms of vocabulary and verbal intelligence (Mill–Hill). A significant age difference was obtained for executive functions (TMT-A, TMT-B, and FAB), with YG being better than AG. Verbal Automatisms scores and IQ-derived from Verbal Automatisms test were significantly higher in AG than in YG. The McNair questionnaire showed more frequent subjective memory complaints in AG than in YG. Table 2b summarizes behavioral results in terms of RT (ms) and %CR for PPTT and for ON and in terms of fluency score (words per minute) for VF (all categories and per category). YG and AG showed a similar level of performance in terms of %CR for PPTT and for ON. AG participants were significantly slower (RT) than were YG during PPTT but not during ON. Moreover, a significant effect of age was observed for VF, with higher scores obtained for YG than for AG.

**Table 2:** Means (average AG; average YG) and standard deviations (SD AG, SD YG) for cognitive (Table 2a)^1^ and behavioral (Table 2b)^2^ data for each group (YG, AG), as well as the statistical values (*t*, p) of differences between groups

	TMT-A	TMT-A	Digit span	FAB	Vocabulary	Fluency	Automatisms	Autom-IQ	McNair
a									
Average YG	7.93	8.75	29.43	17.56	38.62	27.87	29.93	114.62	10.50
Average AG	6.07	5.85	26.28	16.14	39.07	21.28	34.28	125.42	15.28
SD YG	1.65	1.73	5.24	0.62	4.01	11.08	5.76	14.55	5.54
SD AG	1.26	1.35	5.39	1.91	4.02	6.95	2.46	6.19	5.96
*t* value	*T*(28) = 3.43	*T*(28) = 5.04	*T*(28) = 1.62	*T*(28) = 2.08	*T*(28) = 0.30	*T*(28) = 1.92	*T*(28) = 2.61	*T*(28) = 2.57	*T*(28) = 2.27
*p* value	0.001	0.000002	0.12	0.009	0.76	***0*** **.** ***07***	0.01	0.01	0.03
	PPTT-RT	PPTT-%CR	ON-RT	ON-%CR	VF total	VF animal	VF vegetables	VF clothes	VF sports
b									
Average YG	1645	94,6	858	98,4	19.30	24.19	14.94	18.81	19.19
Average AG	2185	92.58	921	98,9	15.30	18.43	13.00	15.14	14.71
SD YG	377	4.67	128	1.71	2.20	3.58	3.56	2.23	2.97
SD AG	332	3.19	107	1.41	2.60	3.87	3.55	4.05	3.22
*t* value	*T*(28) = 4.13	*T*(28) = 1.36	*T*(28) = 1.44	*T*(28) = 0.73	*T*(28) = 3.85	*T*(28) = 4.23	*T*(28) = 1.49	*T*(28) = 3.13	*T*(28) = 3.96
*p* value	0.0003	0.19	0.16	0.47	0.0006	0.0002	0.15	0.004	. 0004

^1^Trail Making Test part A (*TMT-A*) and part B (*TMT-B*); Digit span Memory Test; Frontal Assessment Battery (*FAB*); Vocabulary scale Mill-Hill; Categorical (semantic) Fluency test; Verbal Automatisms test and derived IQ (*Autom-IQ*); McNair Questionnaire.

^2^Accuracy (*%CR*), Latency (*RTs ms*) and Fluency scores: PPTT-RT = Response Time for Pyramid Palm Tree Test (*PPTT*) responses; PPTT-%CR = Accuracy for the Pyramid Palm Tree Test (*PPTT*) responses; ON-RT = Response Time for Object Naming (*ON*) responses; ON-%CR = Accuracy for Object Naming (*ON*) responses; VF total = Fluency score (words per minute) for all categories; VF animal = Fluency score (words per minute) for animal category; VF vegetables = Fluency score (words per minute) for vegetable category; VF clothes = Fluency score (words per minute) for clothes category; VF sports = Fluency score (words per minute) for sport categories

*p* < 0.05 (significant results); *p* = 0.07 (trend to significant for VF)

### Functional MRI results

#### Semantic verbal fluency (VF)

**Fig. 1:**
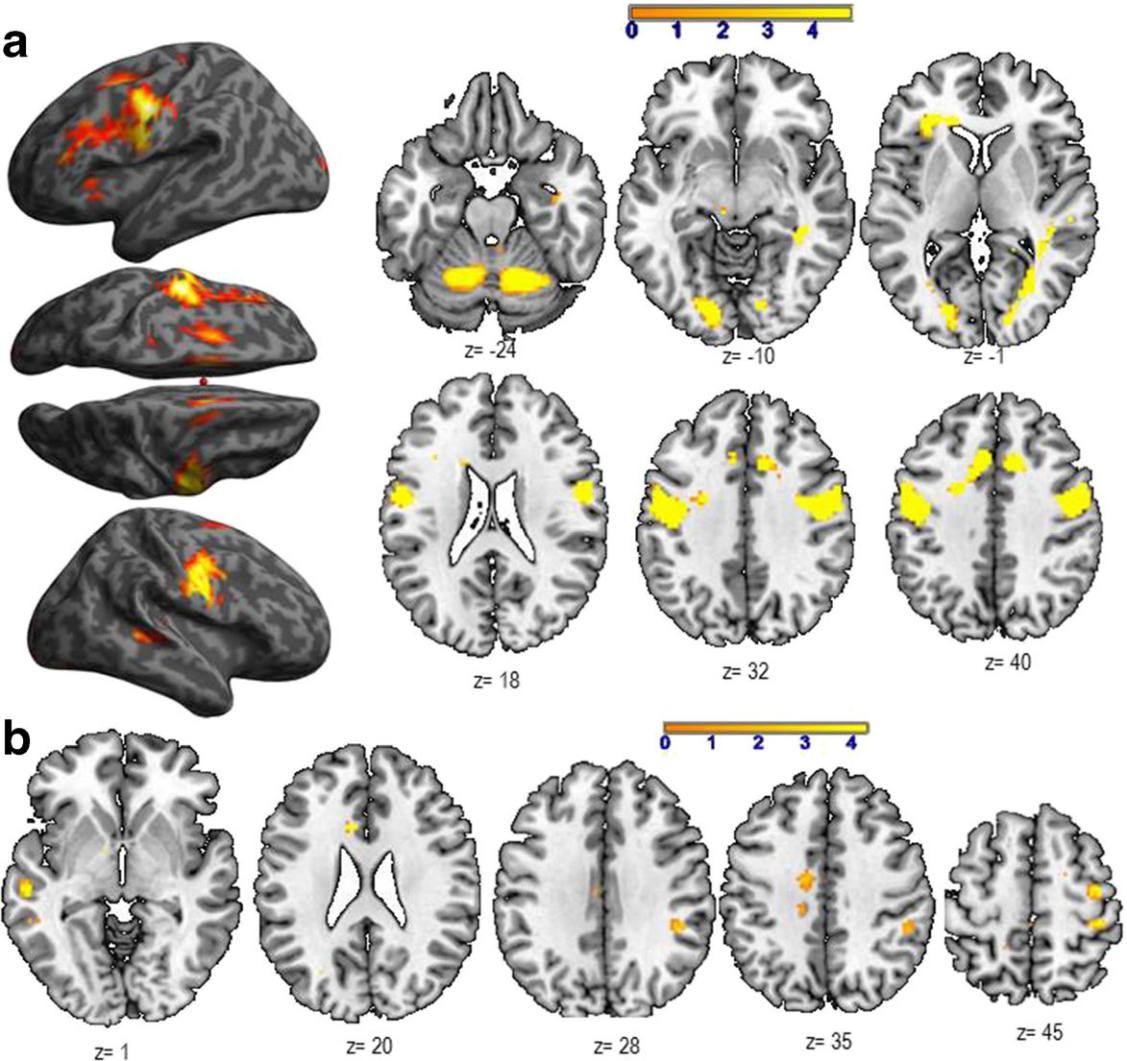
**a** Activated regions during the Verbal Fluency (VF) task (*N* = 30) projected onto 3D anatomical templates and 2D axial slices. **b** Cerebral regions, which are more activated in AG than in YG during VF. The *color scale* indicates the *T* value of the activation. *AG*, aged group; *YG* young group

As illustrated in Table 3a and Fig. 1a, the main effect of the VF task revealed a network mainly composed of frontal regions and the cerebellum. A detailed inspection of Fig. 1a shows that VF also activated occipital, parietal, and temporal cortices, even if the frontal activation remained predominant. The aging effect (AG > YG) (Table 3b and Fig. 1b) was represented by supplemental involvement of the right inferior parietal, left middle-superior temporal, left anterior cingulate, and right motor-sensory regions. No significant activation for YG > AG was obtained for this task. Based on the reported regions for the lexical production (see the “Introduction” section) and highlighted in light blue in Table 3b, we calculated LIs (lateralization indices) for aging-dependent regions. We obtained a positive value (LI = 0.39), suggesting that aging is associated with an overspecialization of the left hemisphere.

**Table 3:**
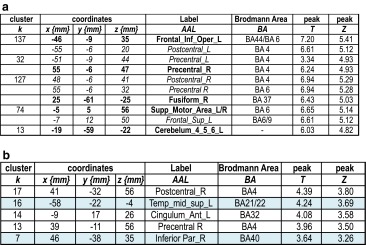
Main effect of Verbal Fluency in terms of peaks of activation for the contrast *Task* vs. *Control* at a group level (one sample *t* test, *N* = 30). Table 3b illustrates the aging effect during Verbal Fluency in terms of peaks of activation for the contrast *Task* vs. *Control* resulting from the two-sample *t* test analysis (AG > YG)

In bold is indicated the most significant voxel of the cluster

Highlighted in light blue are the regions considered to be essential for the lexical production according to the literature. These regions were considered for the calculation of Lateralization Indices (*LI*) to assess the aging effect on the hemispheric specialization. For each peak, we mentioned the number of voxels (*k*), the x,y,z coordinates, the AAL label, the corresponding Brodmann area, and the *T* and *Z* values

*L* left hemisphere, *R* right hemisphere

#### Object naming (ON)

**Fig. 2:**
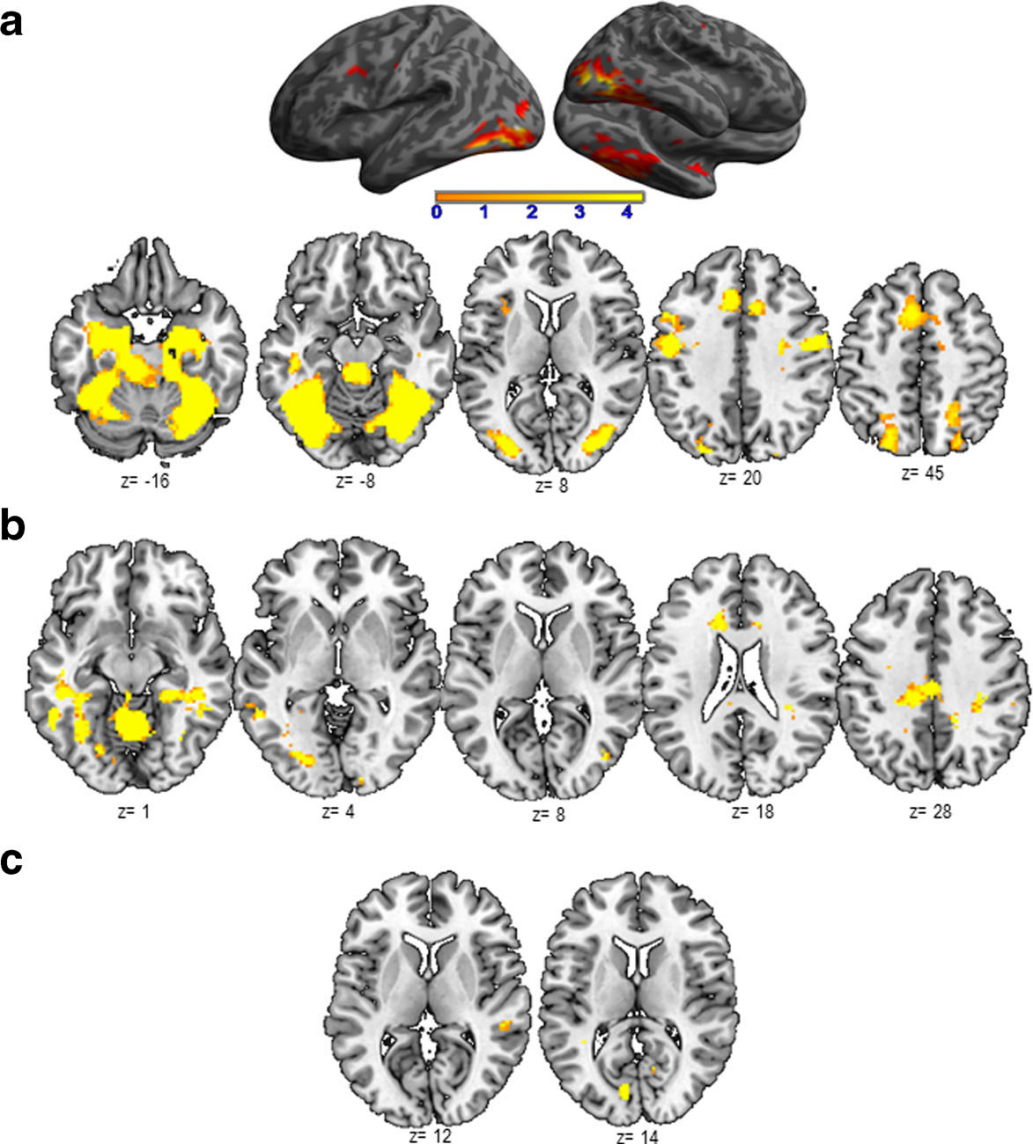
**a** shows activated regions during the Object Naming (ON) task (*N* = 30), projected onto 3D anatomical templates and 2D axial slices. **b** Cerebral regions, which are more activated in AG than in YG during ON. **c** Cerebral regions, which are more activated in YG than in AG during ON. The *color scale* indicates the *T* value of the activation. *AG*, aged group; *YG*, young group

As illustrated in Table 4a and Fig. 2a, the main effect of ON showed a large network, including frontal, parietal, lateral and medial temporal, occipital, and limbic regions. As for the previous task, the bilateral sensory-motor cortices for the articulators were activated as the task was performed overtly. Table 4b and Fig. 2b show that the comparison between the groups revealed a significant aging-dependent activity (AG > YG) in a large network of language regions, with their majority predominant to the left. Several right hemisphere regions, such as the hippocampus and the inferior parietal lobule, were also activated, suggesting supplementary retrieval within the long-term semantic memory, as well as of semantic associations. Other aging-dependent activated regions, such as anterior cingulate and supplementary motor area, might suggest a significant amount of selection and planning in relation with the lexical processing. The LI calculation for language regions highlighted in light blue (Table 4 panel B) suggests that AG recruit more the left hemisphere (LI = 0.62). For this task, we also obtained significant activation for the opposite contrast YG > AG (Table 4c and Fig. 2c), within the superior temporal and left cuneus and without any hemispheric predominance (LI = −0.08).

**Table 4:**
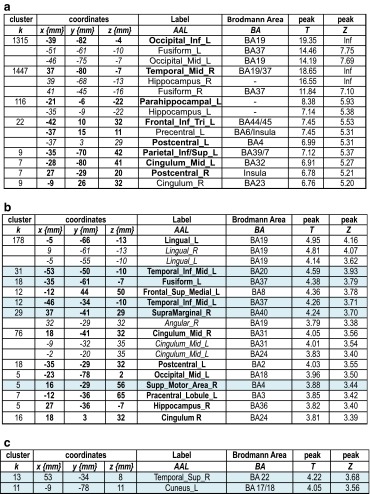
Main effect of Object Naming in terms of peaks of activation for the contrast *Task* vs. *Control* at a group level (one sample *t* test, *N* = 30). Table 4b illustrates the aging effect during Object Naming in terms of peaks of activation for the contrast *Task* vs. *Control* resulting from the two-sample *t* test analysis (AG > YG). Table 4c illustrates activations resulting from the contrast *Task* vs. *Control* for YG > AG and resulting from the two-sample *t* test analysis

In bold is indicated the most significant voxel of the cluster

Highlighted in light blue are the regions considered to be essential for the lexical production, according to the literature. These regions were included in the calculation of Lateralization Indices (LI) to evaluate the aging effect on the hemispheric specialization. For each peak, we mentioned the number of voxels (k), the x,y,z coordinates, the AAL label, the corresponding Brodmann area and the T and Z values

*L* left hemisphere, *R* right hemisphere

#### Semantic categorization Pyramid Palm Tree Test (PPTT)

**Fig. 3:**
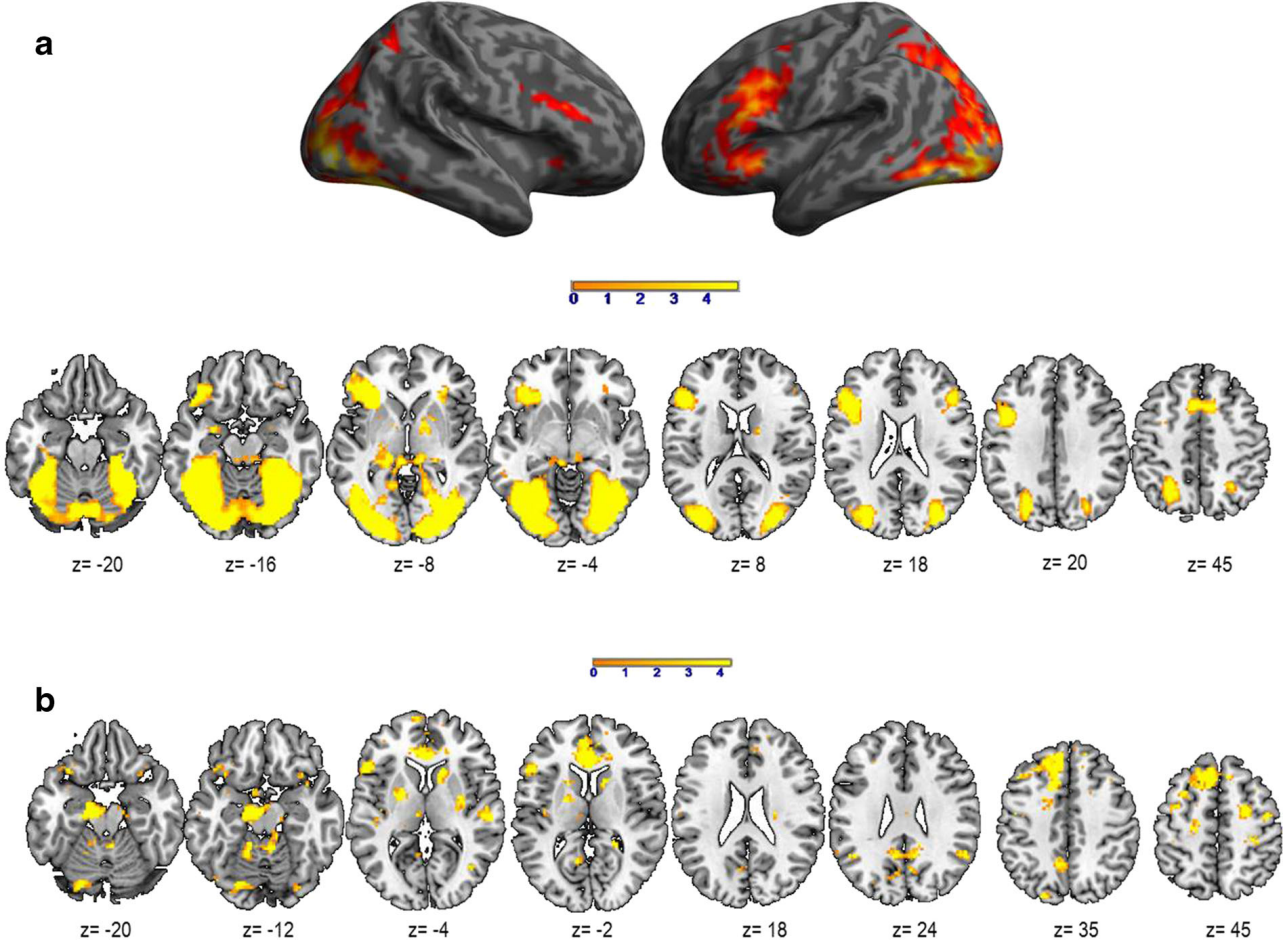
**a** Activated regions during the Semantic Categorization with the Pyramid Palm and Tree Test (PPTT) (*N* = 30) projected onto 3D anatomical templates and 2D axial slices. **b** Cerebral regions, which are more activated in AG than in YG during PPTT. The *color scale* indicates the *T* value of the activation. *AG*, aged group; *YG*, young group

As illustrated in Table 5a and Fig. 3a, the main effect of PPTT revealed a large network of bilaterally activated regions including frontal, parietal, temporal, limbic, and basal ganglia. These regions are generally related to all levels of word retrieval and production, and significant access to conceptual representations and categorization processes. No significant activation was obtained for the opposite contrast YG > AG. The main effect of aging for PPTT (AG > YG) (see Table 5b and Fig. 3b) shows that AG recruited significantly more regions related to all steps of lexical production. LI calculation (regions highlighted in light blue, Table 5b) revealed a positive value (LI = 0.22), suggesting a slight tendency to overactivate the left hemisphere with aging.

**Table 5:**
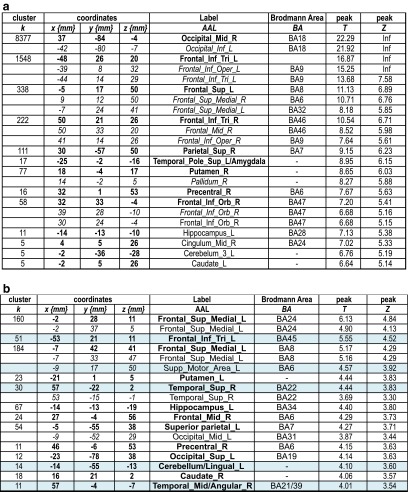
Main effect of Semantic Categorization with PPTT in terms of peaks of activation for the contrast *Task* vs. *Control* at a group level (one sample *t* test, *N* = 30). Table 5b illustrates the aging effect during Semantic Categorization in terms of peaks of activation for the contrast *Task* vs. *Control* resulting from the two-sample *t* test analysis (AG > YG)

In bold is indicated the most significant voxel of the cluster

Highlighted in light blue are the regions considered to be essential for the lexical production according to the literature. These regions were included in the calculation of Lateralization Indices (LI) to evaluate the aging on the hemispheric specialization. For each peak, we mentioned the number of voxels (k), the x,y,z coordinates, the AAL label, the corresponding Brodmann area and the *T* and *Z* values

*L* left hemisphere, *R* right hemisphere

### Correlations between the BOLD signal and the behavioral and neuropsychological scores

**Fig. 4:**
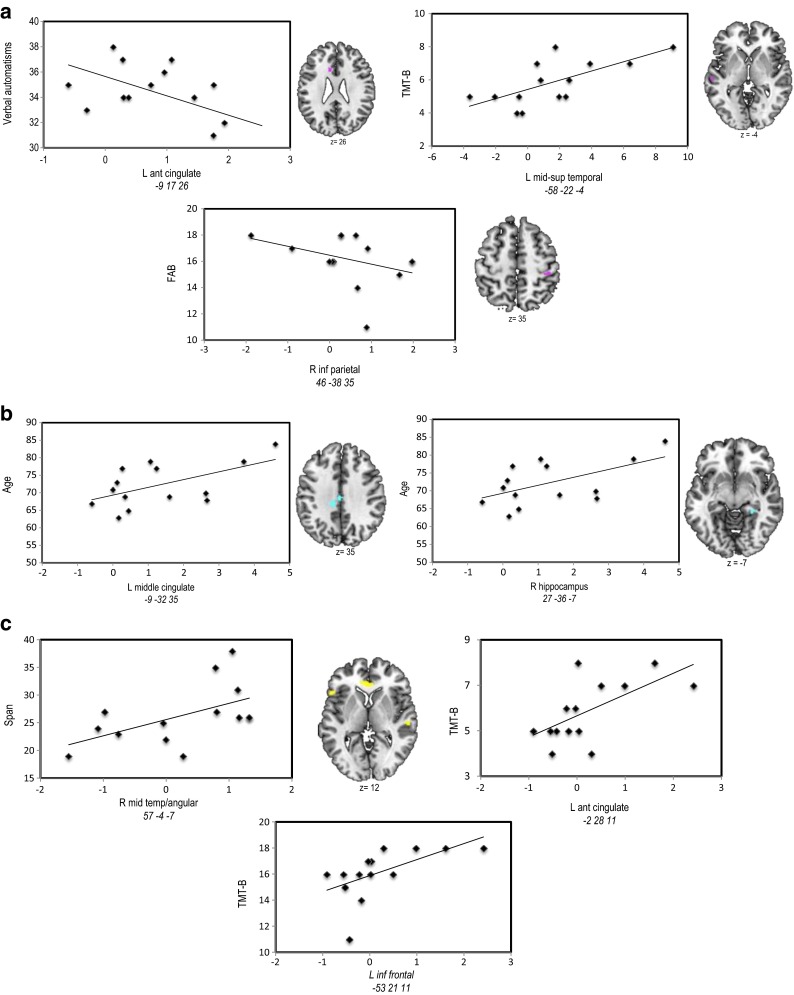
Significant correlation (positive or negative) between the BOLD signal measured in the aging-dependent regions with the neuropsychological scores for Verbal Fluency (VF, **a**) and Pyramid Palm Tree Test (PPTT, **c**) and with Age for Object Naming (ON, **b**). Activated regions were projected onto 2D axial slices and were shown next to the corresponding graphics. *L*, left hemisphere; *R*, right hemisphere

Correlations between the BOLD signal of aging-dependent regions and behavioral scores (response latency for PPTT and fluency scores for VF) did not reveal significant results. Additionally, we performed correlations between the BOLD signal and the neuropsychological scores for each language task (see Table 6a–c). The significant correlations are illustrated in Fig. 4a–c.

**Table 6:**
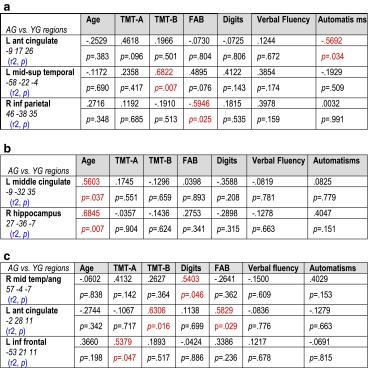
Results (in terms of *r* ^2^ and *p values*) resulting from the correlation analyses performed between the BOLD signal in aging-dependent regions (AG > YG) during Verbal Fluency (Table 6a), Object Naming (Table 6b), and Semantic Categorization PPTT (Table 6c) with Age and with the neuropsychological scores showing aging-effect^1^

The significant correlations are mentioned in red. The *r* negative value indicates negative correlation; positive *r* value indicates positive correlation

^1^Trail Making Test part, *TMT-A* and part B, *TMT-B*; Frontal Assessment Battery *FAB*; Digit span Memory Test; Verbal Fluency test and Verbal Automatisms

Overall, our results showed that the activity (BOLD signal) of aging-dependent regions activated with PPTT and VF tasks, but not with ON task, has been positively correlated with the neuropsychological scores for executive functions, processing speed, and span. This result suggests that PPTT and VF specifically recruit the executive functions and working memory processes associated with RET mechanisms that are involved in word retrieval and generation. Moreover, the activity (BOLD signal) of aging-dependent regions activated with VF correlated negatively with verbal automatisms (left anterior cingulate) and with frontal efficiency battery scores (right inferior parietal), suggesting the necessity to overcome (inhibit) the overlearned lexico-semantic information and automatic processes to generate words. Additionally, ON aging-related regions correlated positively only with age.

## Discussion

The goal of this study was to assess the effect of normal aging on the cerebral correlates of word retrieval and generation. The use of three lexical tasks was expected to cover as large as possible the cerebral networks and psycholinguistic operations involved in lexical production, with a focus on task-specific processes, such as retrieval-executive based (RET) for VF, lexico-semantic representations (SEM) for ON, and both RET and SEM associated with an increased access to conceptual representations and lexico-semantic memory (CON) for PPTT. The hypothesis of this study was that aging does not alter the conceptual level and representations (semantic, phonological) per se, but rather, it induces dysfunction of executive functions related to retrieval abilities for accessing, searching, and selecting concepts and word forms. Operationally, our hypotheses were that the older adults would perform as accurately as the younger participants, but they are slower in terms of response latencies, because of the difficulties to access and activate representations in the context of a decline of executive functions. In terms of cerebral activation, we expected that older adults do not simply recruit less cerebral regions compared to younger, but they show a different pattern of activation, either at the intra- or at the inter-hemispheric level. Correlations between the BOLD signal of aging-dependent regions and the behavioral and neuropsychological scores indicated significant positive and negative correlations for some of the regions. Indeed, positive correlations were thought to reflect compensatory mechanisms to assure a correct level for processing the word retrieval and generation task, by palliating the slowdown of processing speed, flexibility, and frontal efficiency. Negative correlations were also interpreted in terms of compensation, but as a supplementary need to inhibit overlearned unnecessary verbal information and automatic verbal processes, as these processes are significantly predominant in older adults compared to younger participants.

### Neuropsychological results

As illustrated in Table 2a, the results indicate that aging induces a significant decline of executive functions in terms of processing speed, cognitive flexibility, and frontal efficiency, confirmed by a behavioral tendency (without reaching significance) towards a slowdown of lexical fluency rate (VF scores). Executive functions are among the first cognitive processes to decline with aging (Bherer et al. 2004) and particularly under conditions requiring high executive demands (Nielson et al. 2002; Townsend et al. 2006). Decline with aging was observed for working memory, inhibition, and task switching (Rypma and D’Esposito 2000). These processes were differentially involved by the tasks used in our study. Indeed, the working memory seems to be particularly required by the VF and PPTT tasks. Moreover, the flexibility processes (switching, shifting) are mainly necessary for the VF task. Correlations performed between the executive function scores and the BOLD signal of aging-dependent regions revealed significant results for VF and PPTT, but not for ON (see Table 6b). Our interpretation was that compared to VF and PPTT, ON requires a lower amount of executive functions, according to our hypothesis that only VF and PPTT necessitate RET mechanisms. The BOLD signal of aging-dependent regions activated by the ON did not correlate with any neuropsychological score but only with age. This result is coherent with the ON behavioral scores showing that older adults perform as fast as the younger do for this task. Furthermore, verbal automatism scores are significantly more frequent in older than in younger adults, explained by the fact that verbal material learned in early childhood and used throughout life is easily, accurately, and effortlessly recalled, as an automatism. Automatic speech is under the right hemisphere control (Birn et al. 2010) and the negative correlation obtained between verbal automatism scores and the BOLD signal of the anterior cingulate cortex during the VF might suggest that in order to retrieve and select appropriate words to generate, the older adults inhibit the right hemisphere-dependent automatic information (see Table 6a, right inferior parietal) to maintain a correct level of task performance.

### Behavioral scores

Behavioral results (Table 2b) revealed a significant aging effect for fluency rate according to age, in agreement with other results reported in the literature (Clark et al. 2009; Crossley et al. 1997) but in disagreement with some others that found no aging effect (Aine et al. 2006; Bolla et al. 1990; Bolla et al. 1998; Grady 2008) or only a marginal effect (Marsolais et al. 2015). Moreover, behavioral results revealed significant differences between groups in terms of latencies for PPTT, with the older adults being slower than the younger adults. In terms of accuracy, the older and younger participants performed similarly for PPTT and for ON, suggesting no loss of conceptual knowledge. No difference was observed for ON latencies according to age, contrary to other studies (Tsang and Lee 2003) that found a significant aging effect for accuracy and latency during picture naming. The authors explained their results by a greater variability to perform the task for the older than for the younger adults, resulting from different rates of selective changes in cognitive functions among the older adults (Tsang and Lee 2003). Additionally, older participants included in our study were highly educated, explaining, at least partially, the high level of performance for a simple task, such as the object naming. A more difficult task, such as verbal fluency or semantic categorization, requiring supplementary executive resources, might reveal possible weaknesses of lexical production, in agreement with our results. Correlations between the behavioral scores and the BOLD signal in aging-dependent regions did not reveal significant results, suggesting that behavioral measures encompass a large variety of possible cognitive mechanisms and might not detect fine effects, contrary to cognitive scores that are more specifically related to a given cognitive domain.

### Aging effect on cerebral correlates of lexical retrieval and generation

#### Verbal fluency

As illustrated in Table 3a, and more detailed in Fig. 1a, VF activated a network that mainly involved frontal regions, somehow in disagreement with the literature, considering that semantic VF activates not only the left frontal (Fu et al. 2002; Heim et al. 2008; Meinzer et al. 2009, Meinzer et al. 2012) but also the parietal (Birn et al. 2010) and temporal (Birn et al. 2010; Vitali et al. 2005) regions. The left inferior frontal gyrus (IFG) is related to switching between items and categories (Botvinick et al. 2001; Hirshorn and Thompson-Schill 2006) and competition processes among incompatible representations (Thompson-Schill et al. 1999). Overall, the frontal and premotor activation reported in our study could reflect flexibility, inhibition, initiation, and working memory processes (Niendam et al. 2012). SMA is specifically involved in motor and speech production, particularly in the intentional and internally driven behavior involved in our task (self-generated), as suggested by intraoperative electrical stimulation (Duffau et al. 2000; Krainik et al. 2001). neuroimaging (Alario et al. 2006; Picard and Strick 1996; Krainik et al. 2003). and neuropsychological data (Fontaine et al. 2002; Pai 1999). Temporal activation could be explained by the semantic-relatedness of words during VF and can reflect lexical storage, access, and lexical retrieval (Pihlajamaki et al. 2000). The activation of fusiform and lingual gyri is possibly associated with episodic memory retrieval (Gilboa et al. 2004) and with mentally spatial navigation tasks (Maguire et al. 1998). The comparison AG > YG participants (Table 3b and Fig. 1b) revealed aging-related regions predominant to the left (LI = 0.39), including middle-superior temporal cortices, anterior cingulate, and right inferior parietal lobule. Moreover, somato-sensitive and motor areas seem to be additionally recruited by the AG. These results are partially in agreement with other authors (Ansado et al. 2013) who showed bilateral fronto-temporal activation in older participants for VF. Importantly, we did not obtain in older participants supplementary frontal or prefrontal activation during VF. Overall, our fMRI results for the VF task support and additional need with aging to access phonological storage (inferior frontal gyrus) and semantic associations (superior-middle temporal gyrus) in the context of a deficit of cognitive flexibility, as revealed by the significant positive correlation between the TMT-B score and the BOLD signal in the superior-middle temporal gyrus (see Fig. 4a). The supplementary activation of the right inferior parietal lobule could exert an inhibitory role on the non-pertinent semantic overlearned information (negative correlation with the FAB scores, see Fig. 4a), similar to the role of the anterior cingulate gyrus, which is negatively correlated with the verbal automatism scores (see Fig. 4a). Indeed, the cingulate gyrus could be involved in task-switching processing (DiGirolamo et al. 2001) and its negative correlation with verbal scores might suggest more automatic processes needed to shift between items and categories. This could also be interpreted as a different type of compensatory mechanism occurring with aging. In agreement with Ansado et al. (2013) we also suggest that VF difficulties with aging concern mainly the word retrieval within a given category, which becomes more and more effortful over time.

#### Object naming

Our hypothesis was that among the operations involved in lexical production, the ON task specifically recruits the lexico-semantic representations (SEM). As illustrated in Table 4a and Fig. 2a, the activated regions suggest that naming implies several language processes (Bowles 1993) such as the visuo-attentional analysis (occipital), the access to lexico-semantic representations (middle and inferior temporal; inferior parietal), and the activation of phonological label (inferior frontal) before the word production (premotor and sensori-motor) through speaking out. All of these stages are under a cognitive control, attention, and monitoring of executive functions. Our results are in agreement with other studies, indicating the activation of frontal and parietal cortices during naming (Chao and Martin 1999; Kiyosawa et al. 1996). Table 2b and Fig. 2b show that AG recruit supplementary regions and processes, such as the left middle and inferior temporal and fusiform (access to lexico-semantic representations), bilateral occipital (visual analysis), right inferior parietal (semantic processing), left superior frontal (attention and other executive functions), SMA (planning, coordination of output speech), and posterior cingulate gyrus (visual imagery). The supplemental activation of the right hippocampus observed during aging might reflect significant involvement of the retrieval of lexico-semantic representations (Sawrie et al. 2000; Seidenberg et al. 2005). A recent iEEG study (Hamamé et al. 2014) performed during an ON task showed that the hippocampus is involved in finding associations between the identity of an object and its word label. The authors indicated that the latency of the hippocampal response predicts the naming latency, while the inefficient hippocampal activation would be associated with tip-of-the-tongue states. Although the literature findings on the role of the hippocampus in naming concern mainly the left hippocampus, the right activation observed in our study could reflect plasticity mechanisms induced by age with a right-hemisphere shift of activity. The hippocampus is also involved in solving the lexico-semantic ambiguity (Hoenig and Scheef 2005) and in the semantic processing (Binder et al. 2009). These processes are particularly sensitive to aging as suggested by our result showing a significant positive correlation between the hippocampal activity and the age (see Table 6b and Fig. 4b). Another aging-dependent ON region activated in our study was the middle cingulate gyrus (see Table 6b and Fig. 4b). As previously shown (Leech and Sharp 2014). the middle and posterior cingulate gyri modulate the attention focus and have a central role in supporting internally directed cognition as a key node of the Default Mode Network. Contrary to VF and PPTT tasks, the aging-dependent regions revealed by the ON task were not correlated with the executive function scores, suggesting that these processes are not required during naming. These ON aging-dependent regions were correlated only with age, suggesting supplementary effort made by the older adults to correctly perform the task. Additionally, the ON task also revealed two activated regions recruited more by the younger than by the older adult, the right superior temporal and the left cuneus (see Table 4c and Fig. 2c). Their activity (BOLD signal) was correlated neither with cognitive scores nor with age and they (superior temporal and cuneus) might possibly reflect a weaker activation in older compared to younger adults. Overall, the LI based on aging-related regions revealed global left-hemisphere predominance (LI = 0.62) and suggested that aging might increase the left hemispheric specialization.

#### Semantic categorization

The use of the Pyramids Palm Tree Test (Howard and Patterson 1992) was motivated by its clinical use in the standard assessment of semantic memory. This task tests the access to conceptual storage (CON) and lexico-semantic associations (SEM) as well as the recruitment of retrieval mechanisms (RET). Semantic categorization relies on the interaction between language and semantic memory, and our PPTT results (see Table 5a and Fig. 3a) revealed the activation of a large bilateral network predominant to the left, including frontal (left inferior frontal pars *triangularis* and *opercularis*, frontal mid-superior and premotor), temporal (left temporal superior pole, left amygdala and left hippocampus), parietal (right superior parietal lobule), basal ganglia (right striatum), left cerebellum, and right mid-cingulate gyrus. This large cerebral representation might be explained by the representation of concepts, defined by their sensory-motor attributes and features acquired during experience and depending on largely distributed networks for sensory-motor and abstract information (Damasio et al. 1990; Martin and Chao 2001; Warrington and Shallice 1984). In a meta-analysis with studies evaluating the semantic processing, Binder et al. (2009) reported activated areas, which could be grouped into three categories: posterior multimodal and heteromodal association regions, heteromodal prefrontal cortex, and medial limbic regions. In terms of aging effect during PPTT, we showed that older adults recruit supplementary regions (see Table 5b and Fig. 3b), located within the left frontal (inferior, superior, and middle frontal, premotor), left superior parietal, left anterior cingulate, left occipital, right angular, right superior temporal, bilateral cerebellum, and basal ganglia. Part of this supplementary activation in older adults compared to the younger should be interpreted in correlation with the behavioral and cognitive scores and might reflect additional mechanisms recruited by older participants to compensate for the slowdown in retrieving lexico-semantic representations by less efficient executive functions and processing speed. Moreover, the working memory span would be also diminished in older adults and the supplementary activation of the right angular gyrus could reflect a possible compensatory effort to palliate for this deficit. Furthermore, the right angular gyrus could also play a role in the integration of individual concepts into a larger whole context (Binder et al. 2005; Newman et al. 2003) based on semantic associations. Compared to younger participants, older adults could attribute more affective significance to concepts, which is a possible explanation of the supplemental activation of the anterior cingulate gyrus (Mayberg et al. 2014). Taken together, our results obtained with the PPTT task revealed a large cerebral network of aging-related regions located bilaterally, but with a slight predominance to the left. Only some of them compensate for less efficient semantic retrieval (SEM) processes and conceptual (CON) storage in the context of lower executive functions (RET) (see Table 6c and Fig. 4c). Some other aging-dependent regions activated by PPTT in older adults could simply reflect effort, without a specific compensatory role.

For several aging-related regions, the BOLD signal did not correlate with the behavioral or neuropsychological scores, raising the question of their specific role in aging. In fact, these regions could simply reflect dedifferentiation mechanisms, resulting from either a reduced distinctiveness of mental representations and/or an increased neural noise (Li 2002; Li and Lindenberger 1999). Overall, it seems important to better characterize the aging-dependent regions, according to their compensatory role, to maintain the normal execution of the word retrieval and generation. The important aspect that should be clarified is whether the possible language deficits with aging are induced by (a) a direct decline of language operations per se; (b) by an indirect difficulty to access these operations, explained by a slowdown in executive functions; or (c) a direct and non-specific decline of executive functions and processing speed. Given that older adults included in this experiment performed similarly to the younger, we exclude the (a) hypothesis. Moreover, given that the comparison between the older and younger in terms of fMRI data revealed supplemental recruitment of specific language regions and not prefrontal cortices, we suggest that the (c) hypothesis alone does not apply to our data. Finally, the most plausible hypothesis of aging effects described in the current study seems to be (b), suggesting difficulties to perform language operations involved in word retrieval and generation, by a decreased efficiency of executive functions.

This study has several limitations: (a) the number of participants in each group was small additional participants should be included to validate these results; (b) the inclusion of a third supplementary group of participants (less than 30 years of age) would be useful as can provide supplementary information on the language effects of aging; (c) the inclusion in our behavioral results of some supplementary information (such as latencies for VF task) could enrich our data and observations on the aging effect; (d) a higher variability in terms of education and socio-economic level of older participants should be considered, given that older participants included in our study were all highly educated and with high socio-cultural and economic level; this parameter could have biased our results in terms of behavioral and cognitive scores.

## Conclusions

The effect of aging on word retrieval and generation cannot be reduced to a unique mechanism. The normal aging seems to not be associated with conceptual or representational loss but instead with difficulties to access the lexical processes and stages necessary for production of words. This is likely due to a poorer efficiency of the executive functions and processing speed in older adults. Additionally, based on our fMRI results, older participants did not simply activate fewer cerebral regions involved in word retrieval and generation, but they rather showed a different pattern of activated regions at an intra- and inter-hemispheric level. These regions were correlated (positively and negatively) or uncorrelated with the cognitive scores. The calculation of LIs also suggests that the hemispheric specialization during lexical production tends to increase with aging and an overspecialization of the left hemisphere might occur. Finally, we underline the necessity (a) to use a panel of tasks to evaluate the linguistic abilities and to map language regions, and (b) to combine data resulting from a multimodal approach.
